# Elderly at Greater Risk for Root Caries: A Look at the Multifactorial Risks with Emphasis on Genetics Susceptibility

**DOI:** 10.1155/2011/647168

**Published:** 2011-07-06

**Authors:** Daniel Gati, Alexandre R. Vieira

**Affiliations:** Department of Oral Biology, School of Dental Medicine, University of Pittsburgh, Pittsburgh, PA 15260, USA

## Abstract

Root caries is one of the most significant dental problems among older adults today. Many studies have demonstrated that older adults are at greater risk for developing root caries. Here we examine what risk factors older adults are prone to and explain how they contribute to higher rates of oral disease, in particular root caries. The elderly are at risk for root caries due to dentures, lack of dexterity, a shift from complex to simple sugars, and poor oral hygiene. Decreased salivary flow and its manifestations with other social/behavioral and medical factors may provide a more comprehensive explanation to a higher frequency of root caries in older adults.

## 1. Introduction

Due to increasing life expectancy of the dentition, older adults are experiencing root caries and gingival recession, putting them at even higher risk for periodontal disease. Root caries is the major cause of tooth loss in older adults, and tooth loss is the most significant oral health-related negative variable of quality of life for the elderly [1]. Nearly half of all individuals aged 75 and older have root caries [2]. One prominent goal of the dental profession is to preserve and maintain dentitions throughout life. Population projections suggest that the proportion of the population aged 65 years and older will nearly double between 2000 (12.6 percent) and 2030 (20.0 percent), and that the proportion of those aged 85 years and older will increase dramatically over the next 10 to 15 years [3]. This population trend coupled with compelling evidence that people are retaining their teeth into old age suggests that there will be an increased number of older adults with many more natural teeth in the years to come. 

There are known clinical and behavioral risk factors involved in the production and progression of root caries in the elderly. Risks are described in a number of levels, from socioeconomic status to salivary flow to presence of dentures. Data have shown correlations of dietary and oral habits and other variables on root caries [4]. Many risk factors can compromise an older adult's systemic health such as sociodemographic variables, nutrition/diet, and weakened immune system [5]. This paper examines salivary hypofunction, the systemic and oral immune system (immunoglobulins found in saliva) in older adults, and their manifestations. These factors are strongly determined by individual genetic background.

There are several indicators that provide insight into the incidence and prevalence of caries in healthy people and the medical or disability conditions that place individuals at increased caries risk. One indicator is the presence of *Mutans streptococci*, an established etiologic agent for caries activity [6]. One of the main oral behaviors to reduce the amount of bacteria in the oral cavity is regular tooth brushing with a fluoride-containing dentifrice. Inadequate exposure to fluoride accelerates the disease, because fluoride can remineralize decalcified structure. Conditions that compromise good oral hygiene behaviors and oral health are also positively associated with caries risk. These include certain illnesses, physical and mental disabilities, and the presence of existing restorations or oral appliances. Fermentable carbohydrate consumption fuels acid formation and demineralization and is associated with caries, particularly in the absence of fluoride [7]. The amount, consistency, and frequency of consumption determine the degree of exposure. Long-term regular doses of medications containing glucose, fructose, or sucrose may also contribute to caries risk [8].

## 2. Etiology of Caries

Medical conditions such as Sjögren's syndrome, pharmacological agents with xerostomic side effects, and therapeutic radiation to the head and neck lower salivary flow rate to pathological levels and dramatically elevate a patient's risk of caries [9]. This suggests that normal salivary flow rate is protective against caries. Some studies indicate that low buffering capacity, low salivary immunoglobulin A, and low salivary calcium and phosphate may also be linked to increased caries [10].

The inability to maintain good oral hygiene and xerostomia are risk factors of special significance among the elderly, and gingival recession uniquely increases the risk of root caries in elderly populations by exposing previously protected root surfaces to cariogenesis. Low indices of socioeconomic status have been associated with elevation in caries and are also associated with reduced access to care, reduced oral health aspirations, low self-efficacy, and health behaviors that may enhance caries risk [11]. 

Older age is positively associated with the prevalence of root caries [12]. Over half the individuals older than 65 years have experienced root caries [13]. Evidence also suggests that adults who have lived in fluoridated areas throughout most of their lives, including the time of tooth formation, have a lower prevalence of root caries [14]. 

There appear to be a wide variety of risk indicators and risk factors implicated in root caries. These factors include not only oral factors, but also medical, behavioral, and social factors. Caries is an etiologically complex disease process. It is likely that numerous microbial, genetic, immunological, behavioral, and environmental contributors to risk are at play in determining the occurrence and severity of clinical disease. The caries process is endemic and potentially both preventable and curable. Prevention and treatment can be achieved by identifying and arresting or reversing the disease at an early stage. Treatments include application of fluorides, chlorhexidine, sealants, antimicrobials, salivary enhancers, and patient education [11].

## 3. Genetics of Caries

Studies have shown that one's preference to sweet carbohydrates may put one at risk for caries [15]. This preference may be determined by socioeconomic status but also be under genetic control. Studies have examined genetically determined taste sensitivity to 6-n-propylthiouracil demonstrating that individuals with low taste sensitivity experience a lower caries risk than those with high tasting sensitivity [16, 17]. The examination of genetic variation in taste pathway genes (taste receptor, type 2, member 38 (*TAS2R38*), taste receptor type 1 member 2 (*TAS1R2*), and guanine nucleotide-binding protein G(t) subunit alpha-3 (*GNAT3*)) and their relation to caries revealed some associations. *TAS1R2* is a member of sweet taste receptor family, and *GNAT3* codes for the G protein gustducin, which mediates taste receptor signaling in the taste buds of the lingual epithelium. A significant association was found for certain alleles in *TAS2R38* that were protective from caries, while other haplotypes were associated with caries risk. This association held true only for the primary dentition with individuals with a mean age of 3.4 years. There was no significant association in the mixed and permanent dentitions, which had individuals with mean ages of 9.8 and 29.4 years, respectively. The *TAS2R38* single nucleotide polymorphisms that were found to be protective for caries cause amino acid changes in the taste receptor that are associated with bitter sensitivity [18]. Variations of the genetic makeup of these genes may contribute to differences in dietary habits that influence the caries risk of these children. Evaluation of children classified with different tasting abilities has also been associated with body weight and dietary habit differences [19]. At this point, these studies have not targeted the elderly and/or root caries.

To support the notion that caries is a disease with a genetic component, one study used DNA samples collected from 110 individuals older than 12 years of age from Guatemala and documented who had a higher or lower caries experience using DMFT (Decayed, Missing due to caries, Filled Teeth) scores [20]. Enamel proteins such as ameloblastin and tuftelin are associated and crucial for proper enamel formation. Single-nucleotide polymorphism markers were genotyped in selected candidate genes (*ameloblastin*, *amelogenin*, *enamelin*, *tuftelin-1*, and *tuftelin interacting protein 11*) that influence enamel formation. Having at least one copy of the rare *amelogenin* marker allele was associated with increased age-adjusted caries experience. This association was stronger in individuals with higher DMFT (DMFT ≥ 20; *P* = .0000001), suggesting that a variation in *amelogenin* may contribute to caries susceptibility in the population studied. These results were confirmed in an independent cohort from Turkey [21]. 

Besides genes related to taste preferences and enamel formation, immune response genes have also been considered as candidate genes to caries susceptibility. Three single nucleotide polymorphisms in *DEFB1* (beta defensin 1) were tested in a cohort of unrelated adult individuals [22]. Carrying a copy of the variant allele of the *DEFB1* marker rs11362 increased the DMFT and DMFS scores more than fivefold. Also, carrying a copy of the variant allele of the *DEFB1* marker rs179946 correlated with low DMFT scores. A high caries experience promoter haplotype (GCA) increased DMFT scores twofold, and a low caries experience promoter haplotype (ACG) decreased DMFT scores two-fold. These results suggest that functional polymorphisms of *DEFB1* are potential markers for caries.

## 4. Saliva and Immure Response

### 4.1. Salivary Flow

One critical contribution to oral health is adequate salivary flow. Saliva contains many chemicals that keep the oral cavity healthy. In healthy individuals, salivary flow tends to remain stable from younger to older ages [23]. As we age our immune system weakens and fewer antimicrobial immunoglobulins are produced and found in saliva [24]. Medications that are prescribed to the elderly in fact can cause impaired salivary flow with no change in the immune system [25]. Many medications, chemotherapy, radiation treatments, and some diseases can decrease salivary gland function and therefore make caries and other oral diseases more likely to occur. Some common drugs that may cause dry mouth are high blood pressure drugs, cholesterol lowering drugs, pain medications, muscle relaxants, allergy, and asthma medications. No matter what the cause, it is undisputed that saliva is essential in neutralizing the acidic environment, thus inhibiting the growth of bacteria. Any decreased levels of saliva can put one at increased risk for developing caries.

### 4.2. Genetic Contributions of Immunoglobulins and Salivary Proteins

When studying the elderly population, researchers have looked at age-related differences in whole and parotid saliva secretion related to the production of saliva, as well as the immune factors in saliva. The levels of serum immunoglobulin G (IgG) and IgM were significantly reduced in older individuals, whereas no significant reduction in the level of IgA with age was observed [26]. No significant changes in any immunoglobulin levels with age were found in parotid saliva, but significant reductions in the secretion rates of IgA and IgM, but not IgG, in whole saliva were detected in the oldest age individuals. The results demonstrate a decline in immunoglobulin concentrations with increased age, which may contribute to the increased susceptibility of elderly individuals to oral diseases. 

Age-related differences in saliva gene expression have been noted in mice [27]. One hundred and sixty of the 1,328 parotid gland genes show more than a twofold change in expression. The majority of these genes (96%) exhibited decreased expression in elderly mice. These genes are associated with numerous biological pathways. The effects of age on specific gene expression in the human parotid gland may provide insight into functional and morphological changes in the oral cavity and its associations to oral disease. 

When examining unstimulated and stimulated submandibular/sublingual saliva flow rates, unstimulated and stimulated parotid saliva flow rates, and different proteins (lactoferrin, secretory IgA, albumin, lysozyme, mucin, and cystatin), significant associations were found between caries, age, and specific individual submandibular/sublingual salivary protein levels. These changes in salivary components during aging were correlated with high caries prevalence. Therefore, these changes in saliva components over age may represent caries risk indicators [28]. 

Age has a significant influence on the expression of genes associated with reduced protein biosynthesis of salivary gland secretion. Specific genes may be most affected by aging [29]. The expressions of both *HLA-DQA1* (major histocompatibility complex, class II, DQ alpha 1) and *HLA-DQB1* (major histocompatibility complex, class II, DQ beta 1), genes involved in immuneresponse, were decreased in the parotid gland in the elderly. Chemokines attract neutrophils and promote their adherence to endothelial cells. Chemokine ligand 10 (*CXCL10*) also showed lower expression in the aged population. Several other proteins known to be involved in different immune response pathways showed altered expression in aged population (e.g., *IRF1*, *IRF7*, *GBP1*, *IFITM1*, *IFITM2*, *IFITM3*, *PSMB8*, and *PSMB9*). Complex remodeling of the immune system occurs during aging, which may contribute significantly to systemic diseases in the elderly. Diseases such as infections, autoimmune, and neoplastic pathologies that aged individuals are particularly susceptible to involve dysregulation of immune function. The number of elderly is dramatically increasing, and consequently, geriatric pathology is becoming a more important aspect of clinical practice. In light of this, salivary gland function may prove to be a risk factor worth evaluating in the elderly. 

## 5. Brief Discussion on Other Risk Factors

### 5.1. Diet

Diet is a very important factor in preventing caries since certain foods and snacks can greatly increase the number of bacteria that forms the decay-causing plaque. The more sweetened snacks consumed and the more frequently they are consumed increase the risk for developing caries. The frequency of sugar intake is more important than the amount of sugar consumed in the development of caries [30]. Therefore, minimizing snacking is recommended since snacking creates a continual supply of nutrition for acid-creating bacteria in the mouth. Also, chewy and sticky foods (such as dried fruit or candy) tend to adhere to teeth longer and consequently are best eaten as part of a meal. 

When studying the elderly population, it is beneficial to look at other factors such as diet, which together with decreased salivary flow make one more susceptible to root caries. When dietary habits, microbial factors, and salivary factors were analyzed together in older adults who had root caries compared to adults who did not have root caries, individuals with root caries ate a greater number of meals a day and had higher sugar intake [31]. Root caries subjects had significantly higher lactobacilli counts and less salivary buffering capacity suggesting that higher microbial counts and less salivary flow may be risk factors associated with root caries in older adults. 

### 5.2. Bacteria

Plaque consists of bacteria and an extracellular matrix that contains lipids, proteins, and polysaccharides. Teeth are more vulnerable to an increase in bacterial plaque when carbohydrates in the food are left on teeth after every meal. In the presence of sugar and other carbohydrates, bacteria in the mouth produce acids that can demineralize enamel, dentin, and cementum. The more frequently teeth are exposed to this environment, the more likely caries are to occur. The bacterial profiles associated with root caries in elderly subjects exhibit reduced diversity [41]. Certain bacterial species appear to be strongly associated with health, as they are rarely detected or are absent from root caries carriers but are commonly found in healthy subjects. In root caries, *Veillonella parvula, Veillonella dispar*, *Selenomolas noxia*, *Campylobacter gracilis*, *Streptococcus mutans*, *Selenomonass putigena,* and *Fusobacterium nucleatum* are found at high levels. *Lactobacilli* appears to be associated with disease, as they are common in carious lesions, while rare or absent in healthy teeth. In individuals with no caries, *Streptococcus mutans* are less common and *lactobacilli* are absent, while for individuals with root caries, levels of *Streptococcus mutans* and *lactobacilli* are increased. 

The prevalence of *Streptococcus mutans* alone or in combination with *lactobacilli* is similar in root caries lesions. *Lactobacilli* are absent in healthy subjects but highly present in carious dentin, supporting the suggestion that *lactobacilli* might play a significant role in the progression of root caries. Bacterial species typically associated with root caries can be detected, such as *Streptococcus mutans*, *lactobacilli*, and *Actinomyces* [41].

### 5.3. Oral Hygiene

Oral hygiene is a major component of oral disease susceptibility. The relationship between oral health and oral behaviors is widely recognized [42]. Although many variables influence the production and progression of oral disease, the one variable that shows an immediate and long lasting significant effect on one's oral health is oral hygiene. The purpose of oral hygiene (brushing and flossing daily) is to minimize, remove, and prevent the formation of plaque. Efficient oral hygiene practices have positive effects on root caries [43].

Adjuvants of oral hygiene have also been evaluated in regard to root caries. Three monthly applications of chlorhexidine-thymol varnish (Cervitec) over one-year limits the progress of existing root caries lesions and reduces the incidence of root caries [44]. When fluoride varnish, 1% chlorhexidine, 40% chlorhexidine, and professional tooth cleanings were compared in regard to root caries, all methods showed significant reduction in the amount of microbiota (bacteria). These data suggested that tooth cleaning alone might be as effective in reducing plaque formation (and subsequently root caries) as fluoride or chlorhexidine [45]. 

### 5.4. Systemic Diseases

One of the more groundbreaking studies of oral disease today is examining the associations between oral and systemic diseases. Data from the National Health and Nutrition Examination Survey 1999–2004 showed that individuals with rheumatoid arthritis, diabetes, or a liver condition were twice as likely to have an urgent need for dental treatment [46]. The data also showed that arthritis, cardiovascular diseases, diabetes, emphysema, hepatitis C, obesity, and stroke were all associated with dental disease. Unmet dental care needs were observed among participants with chronic diseases. These results suggested that some chronic diseases increase the risk of developing dental disease. Others may interpret this association as meaning that those with systemic disease tend to neglect their oral health and so show a higher incidence of oral disease.

In an attempt to evaluate whether self-reported systemic diseases were associated with caries experience, data from the University of Pittsburgh School of Dental Medicine Dental Registry and DNA Repository regarding medical history and caries experience (DMFT and DMFS; Decayed, Missing due to caries, Filled Teeth/Surface) were analyzed [47]. An association was found between higher caries experience (DMFT above 15 and DMFS above 50) and asthma and epilepsy.

Cardiovascular diseases have also been associated with higher caries experience, particularly in individuals 80 years or older [48]. Individuals with three or more active root caries lesions have more than twice the odds of cardiac arrhythmias than ones without active root caries. These results did not notably change after adjusting for age, medications that reduce saliva, and number of teeth. The findings indicate that there may be a link between active root caries and cardiac arrhythmias in those aged 80 and older. One explanation for these findings is that both cardiac arrhythmias and caries are simply markers of declining general health. 

Xerostomia, commonly associated with oral disease, has also been associated with type 2 diabetes mellitus [49]. The prevalence of xerostomia is higher (62%) in subjects with type 2 diabetes mellitus in comparison to the nondiabetic controls (36% prevalence; *P* = .001). In the same way, the prevalence of hyposalivation is higher in individuals with type 2 diabetes mellitus (46%), whereas only 28% of the controls had hyposalivation (*P* = .03). Subjects with hyposalivation had significantly higher numbers of *mutans streptococci*, *Lactobacillus*, and *Candida* in the saliva compared to those without hyposalivation. The higher number of pathogens and decreased salivary flow may very well explain why diabetics have or are at higher risk for oral disease.

There are several review papers dealing with risk factors related to root caries. These papers revisit aspects related to diet, microbial colonization, oral hygiene, and concomitant systemic illnesses, as well as several topics not covered in this section, such as nonimmunoglobulin salivary agents, chewing ability, sugar clearance, antimicrobial mouthwashes, saliva substitutes, and sugar substitutes. For further information on these areas, we suggest the reader to consult papers [1, 32–40]. 

## 6. Conclusion

As the US population ages, and more teeth are retained, there will be a higher prevalence of root caries and untreated dental decay. Therefore, the demand for dental services in the population of the oldest elderly people is likely to increase. The evaluation of a cohort of elderly aged 79 years or older (mean age 85.1 years) with a mean of 19.4 remaining teeth showed that nearly all subjects (96 percent) had coronal decay experience and nearly two-thirds (64 percent) of the individuals had root caries experience, with 23 percent having untreated root caries. Utilization of dental services was high among the dentate elderly, with nearly three-quarters reporting having visited a dentist within the past year [50]. Those with active coronal or root decay are more likely to be male and to have a history of tobacco use; they are less likely to have visited a dentist within the past year or report regular use of dental services. 

The most recent look at caries frequency clearly indicates a marked increase in the prevalence of caries [51]. This global increase in caries prevalence affects all individuals and all surfaces of teeth. There are a wide variety of risk factors associated with the development of caries, and although there are differences of opinion regarding the cause of the increase in caries it should be agreed upon that public health strategies are needed to renew the fight against caries and promote prevention of future oral disease. Awareness and promotion of water fluoridation, fluoride applications, emphasis on proper tooth brushing with a fluoride dentifrice, flossing, a proper diet, and regular dental office visits can hinder the progression of future caries and can result in an increase in the oral health of all individuals. More programs such as school oral health educational programs are needed to benefit and enhance the oral health (and systemic health) of individuals worldwide. 
